# Dysregulation of Glypicans and Notum in Osteoarthritis: Plasma Levels, Bone Marrow Mesenchymal Stromal Cells and Osteoblasts

**DOI:** 10.3390/cells13100852

**Published:** 2024-05-16

**Authors:** Irene González-Guede, María López-Ramos, Luis Rodríguez-Rodríguez, Lydia Abasolo, Arkaitz Mucientes, Benjamín Fernández-Gutiérrez

**Affiliations:** 1 UGC de Reumatología, Hospital Clínico San Carlos, IdISSC, 28040 Madrid, Spain; igguede@salud.madrid.org (I.G.-G.); maria.lopez.ramos@salud.madrid.org (M.L.-R.); lrrodriguez@salud.madrid.org (L.R.-R.); lydia.abasolo@salud.madrid.org (L.A.); arkaitz.mucientes@salud.madrid.org (A.M.); 2 Facultad de Medicina, Universidad Complutense de Madrid, 28040 Madrid, Spain

**Keywords:** osteoarthritis, bone marrow mesenchymal stem cells, osteoblast, glypican, notum

## Abstract

In this study of the alterations of Glypicans 1 to 6 (GPCs) and Notum in plasma, bone marrow mesenchymal stromal cells (BM-MSCs) and osteoblasts in Osteoarthritis (OA), the levels of GPCs and Notum in the plasma of 25 patients and 24 healthy subjects were measured. In addition, BM-MSCs from eight OA patients and eight healthy donors were cultured over a period of 21 days using both a culture medium and an osteogenic medium. Protein and gene expression levels of GPCs and Notum were determined using ELISA and qPCR at 0, 7, 14 and 21 days. GPC5 and Notum levels decreased in the plasma of OA patients, while the BM-MSCs of OA patients showed downexpression of GPC6 and upregulation of Notum. A decrease in GPC5 and Notum proteins and an increase in GPC3 were found. During osteogenic differentiation, elevated GPCs 2, 4, 5, 6 and Notum mRNA levels and decreased GPC3 were observed in patients with OA. Furthermore, the protein levels of GPC2, GPC5 and Notum decreased, while the levels of GPC3 increased. Glypicans and Notum were altered in BM-MSCs and during osteogenic differentiation from patients with OA. The alterations found point to GPC5 and Notum as new candidate biomarkers of OA pathology.

## 1. Introduction

In a global context, Osteoarthritis (OA) shows a dynamically increasing prevalence related to the increase in risk factors such as obesity and an aging population [1]. This disease of the joints affects the articular cartilage, subchondral bone, ligaments and synovial membrane [2]. The most characteristic symptoms of OA include pain, stiffness, reduced movement, swelling and joint crepitation [3].

Currently, there are no effective medications to treat Osteoarthritis. The most commonly used pharmacological options are analgesics to relieve pain and, in more advanced cases, joint replacement surgery [4]. Nowadays, OA is a challenge that is prioritized by healthcare systems. Given the high prevalence and disability of patients with Osteoarthritis, it is essential to find drugs that can minimize its effects.

Mesenchymal stem cells (MSCs) are cells that have the ability to differentiate into cells of the mesodermal lineage, such as chondrocytes, osteocytes and adipocytes. In adults, MSCs reside in the bone marrow and can be obtained after in vitro proliferation [5]. In joints, two main cell types can be obtained from MSCs: chondrocytes and osteoblasts. In mesenchymal stem cells from the bone marrow of patients with Osteoarthritis, it has been proven that osteogenic potential is increased and chondrogenic and adipogenic potential is attenuated compared to those of healthy donors. MSCs differentiate preferentially into osteoblasts and significantly less often into chondrocytes, causing a breakdown in homeostasis between osteogenesis and chondrogenesis. These facts implicate MSCs in the increased bone mass and cartilage loss that characterize OA [6]. Along these lines, several studies have focused on alterations in bone metabolism as a direct contributor to OA and their influence on cartilage injuries. Several signaling pathways, growth factors, cytokines and other components related to bone formation and maintenance have been found to be altered in OA. This evidence, together with the fact that abnormal activity in the subchondral bone is observed in the early phase of OA, hints at the possibility that pharmacological interventions aimed at regulating bone metabolism can improve or preserve the joint structure. In fact, new therapeutic proposals for Osteoarthritis have focused on bone remodeling and show promising results [7].

One of the most important signaling pathways related to bone formation and maintenance is the Wnt/β-catenin pathway. Wnts are essential in the differentiation, proliferation and synthesis of the bone matrix which osteoblasts perform [8]. The Wnt/β-catenin signaling pathway is a normal physiological response to mechanical loading in bone [7]. Several studies show that the Wnt signaling pathway determines the cell fate of mesenchymal cells, as it favors the formation of osteoblasts and inhibits the formation of chondrocytes and adipocytes while maintaining the balance between bone formation and bone resorption [9].

Several studies have demonstrated that the Wnt/β-Catenin signaling pathway is involved in the pathology of Osteoarthritis. An accumulation of β-catenin has been found, suggesting the excessive activation of this signaling pathway [10]. In in vitro assays, it has been proven that alterations in the expression of components of the Wnt pathway occur during osteogenesis [11]. Messenger RNA levels of WISP-1, a gene associated with the Wnt/β-Catenin pathway, are overexpressed during OA and are capable of inducing cartilage damage [12]. Blockading of canonical Wnt signaling prevents the hypertrophic differentiation of chondrocytes [13]. All these findings show that alterations related to this signaling pathway contribute to the damage and loss of articular cartilage and the stimulation of bone formation in OA.

Proteoglycans, specifically Glypicans, play a fundamental role in the stimulation of the Wnt pathway. Glypicans are an important family of heparan sulfate proteoglycans that are attached to the outer surface of the plasma membrane via a glycosylphosphatidylinositol (GPI) linkage. This protein family contains six members (GPCs 1 to 6) in the human genome that share a characteristic domain of 14 conserved cysteine residues. Due to their similarity, the Glypicans are divided into two subfamilies: GPCs 1, 2, 4 and 6 (with a 35–63% homologous sequence) and GPC3 and GPC5 (54% homologous). The primary function of membrane-bound Glypicans is to regulate the signaling of Wnts, Hedgehogs (Hhs), fibroblast growth factors (FGFs) and bone morphogenetic proteins (BMPs). Their stimulatory mechanism is based on the ability of Glypicans to facilitate and/or stabilize the interaction of Wnts with their signaling receptors. In addition to being anchored to the cell membrane, Glypicans can be found in the extracellular environment and do not interact with the regulation of the Wnt pathway [14,15]. In any case, more studies are needed to achieve an in-depth understanding of the mechanisms of each of the Glypicans, both those bound to the membrane and those found in soluble form.

Until relatively recently, it was assumed that Glypicans were released from the membrane by Notum. Notum is primarily a negative feedback enzyme of Wnt signaling. For this reason, it was believed that Notum could act as a phospholipase enzyme and cleave the GPI anchor, which keeps Glypicans attached to the cell membrane [16]. However, a recent study by Kakugawa et al. studied the role of Notum in depth and attributed other functions to it [17]. As they demonstrated, Notum is a carboxylesterase that eliminates an essential palmitoleate fraction from Wnts and deactivates the signaling pathway. For their part, Glypicans direct Notum to bind with Wnts, since a glycosaminoglycan binding site was found in Notum.

We have studied the dysregulations that may exist in Glypicans 1 to 6 and Notum in Osteoarthritis patients at the cellular and circulating levels. Bone marrow mesenchymal stem cells from patients diagnosed with Osteoarthritis and healthy donors were studied in vitro. Additionally, osteogenic differentiation was induced to determine if there were alterations in the osteoblasts, and to evaluate whether any alterations were primarily intracellular, extracellular or both.

## 2. Materials and Methods

### 2.1. Patients

Written informed consent was obtained from all subjects before sample collection. The study protocol was approved by the institutional ethics committee (Comité Ético de Investigacion Clínica Hospital Clinico San Carlos—Madrid, Spain) in accordance with the principles of the Declaration of Helsinki.

### 2.2. Plasma

Twenty-five patients with OA were recruited according to the American College of Rheumatology (ACR) criteria alongside twenty-four control subjects belonging to the biobank of our institution. Blood samples were collected in K2-EDTA tubes and centrifuged at 2000× *g* for 15 min; aliquoted plasma was stored at −80 °C.

### 2.3. Bone Marrow Mesenchymal Stromal Cell Isolation

BM-MSCs were prepared from the bone marrow aspirates of eight Osteoarthritis patients undergoing joint replacement and eight healthy donors with subcapital hip fractures. BM-MSCs were isolated using density gradient centrifugation (Ficoll-Paque, GE Healthcare, Madrid, Spain) and subsequently expanded in DMEM with Glutamax (Gibco, 21885, Madrid, Spain), 10% FBS and 1% Pen/Strep (Gibco), subsequently referred to as the “culture medium”. The obtained MSCs were confirmed using flow cytometry and histochemistry assays, as we have previously described [11], to determine whether they satisfied the minimal criteria for the definition of MSC proposed by the International Society for Cellular Therapy. Antibodies used were mouse anti-human IgG1 against CD73, CD90 and CD105, CD14, CD34 and (rat anti-human IgG2b) CD45. All the antibodies and isotype controls were R-phycoerytrin (PE)-conjugated (Miltenyi Biotech, Madrid, Spain). Immunostaining was performed with incubation for 30 min at 4 °C. After washing, cells were fixed with 0.1% paraformaldehyde prior to analysis in a “Gallios” flow cytometer (Beckman Coulter, Madrid, Spain).

### 2.4. Cell Culture

Mesenchymal stem cells were cultured in 6-well plates. After 24 h in the culture medium, the MSCs and supernatant were collected from one of the wells at a basal value *t* = 0. One portion of the BM-MSCs was left in the culture medium, while the other portion was added to an osteogenic induction medium (StemMACS Osteodiff Media Human, 130-091-678, Mitenyi Biotec, Madrid, Spain) with 1% Pen/Strep (Gibco). Cells in both conditions were cultured for 21 days. The supernatant was collected at 7, 14 and 21 days. BM-MSCs and osteoblasts were harvested and preserved in RNAlater (Invitrogen, Madrid, Spain) on the same days. The supernatant and cells were stored at −80 °C.

### 2.5. Determination of Glypicans and Notum in Plasma and Cell Culture Supernatants

Glypican and Notum levels were determined using commercial kits and following the manufacturer’s instructions (Human Glypican 1 ELISA kit, ref. EH222RB, Invitrogen; Human Glypican 2 ELISA kit, ref. E-EL-H1711, Elabscience; Human Glypican 3 ELISA kit, ref. EH223RB, Invitrogen; Human Glypican 4 ELISA kit, ref. E-EL-H1713, Elabscience; Human Glypican 5 ELISA kit, ref. EH224RB, Invitrogen; Human Glypican 6 ELISA kit, Ref. CSB-EL009708HU, CUSABIO; Human NOTUM ELISA kit, ref. EH1816, FineTest). The absorbance was measured at 450 nm (Heales MB-580, Shenzhen Heales Technology Development Co., Ltd., Shenzhen, China).

### 2.6. mRNA Analysis

The RNA isolation of cells was performed using the Speedtools Total RNA Extraction Kit (Biotools, Madrid, Spain) and concentration was measured using a spectrophotometer (NanoDrop One, Thermo Scientific, Madrid, Spain). To obtain cDNA, the Maxima First Strand cDNA Synthesis Kit for RT-qPCR (ThermoScientific) was used. The TaqMan^®^ Universal PCR Master Mix kit (Applied Biosystems, Madrid, Spain) was used to perform the qPCR on a 7500 Fast Real-Time PCR System (Applied Biosystems).

The expression of GPC1 (Hs00892476_m1), GPC2 (Hs00415099_m1), GPC3 (Hs01018936_m1), GPC4 (Hs00155059_m1), GPC5 (Hs00942154_m1), GPC6 (HS01569271_m1) and NOTUM (Hs00394510_m1) was evaluated. Relative gene expression was normalized to the housekeeping genes β-actin (Hs99999903_m1) and 18S (Hs99999901_s1). The data were calculated with the fold change method (2^−ΔΔCt^).

### 2.7. Statistical Analysis

All experiments were analyzed with GraphPad Prism 8.0. For the detection of outliers, the ROUT method (Q = 1%) was used. If the data followed a normal distribution, they were analyzed with a *t*-test; when the data did not follow a normal distribution, the non-parametric Mann–Whitney test was performed. A significance level *p* < 0.05 was considered significant. Biochemical data are expressed as mean ± SEM.

## 3. Results

### 3.1. Circulating Levels of Glypicans and Notum

#### 3.1.1. Demographic Characteristics of the Study Population

To study the levels of Glypicans and Notum in plasma, 25 patients with Osteoarthritis and 24 healthy donors were recruited. The mean age (±SD) ranged between 61.56 (±7.70) in OA patients and 58.21 (±4.40) in healthy donors. The age between groups was not statistically different when evaluated with the *t*-test (*p* = 0.0692).

Of the OA patients, 22 were women and 3 men, and the healthy donors included 19 women and 5 men. No differences were identified between the groups regarding gender when the Chi-square test was applied (*p* = 0.4635).

#### 3.1.2. Plasma Levels in Controls and Patients with Osteoarthritis

**Figure 1:**
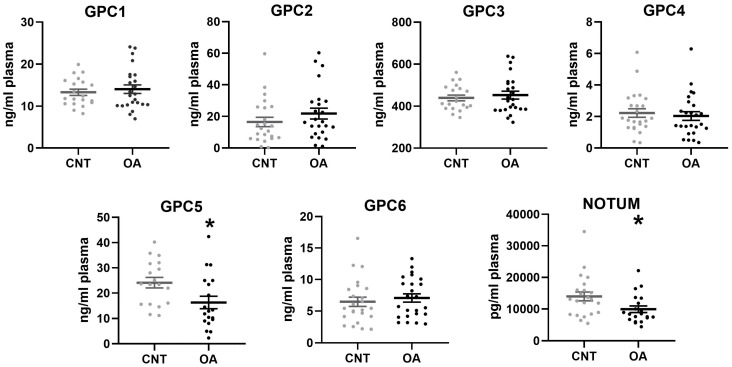
Plasma levels of Glypicans and Notum in patients with Osteoarthritis (*n* = 25) and controls (*n* = 24). Non-parametric Mann–Whitney test (GPC2, GPC4, GPC6 and Notum) and *t*-test (GPC1, GPC5, GPC3) were used to compare the groups: * *p* < 0.05. All bar graphs are presented as mean ± SEM. A decrease in GPC5 and Notum were found in OA patients.

Patients with OA showed a significant decrease in plasma levels of GPC5 (*p* = 0.0208) and Notum (*p* = 0.0260) compared to healthy controls. No differences were found between the two groups for the rest of the Glypicans (Figure 1).

### 3.2. BM-MSCs and Osteoblasts

Five women and three men were recruited from each group, with a mean age of 72.50 ± 8.04 in patients with OA and 69.50 ± 10.30 in controls. The two groups did not differ in age when evaluated with a *t*-test (*p* = 0.5264).

#### 3.2.1. Gene Expression of MSCs during Osteogenic Differentiation

**Figure 2:**
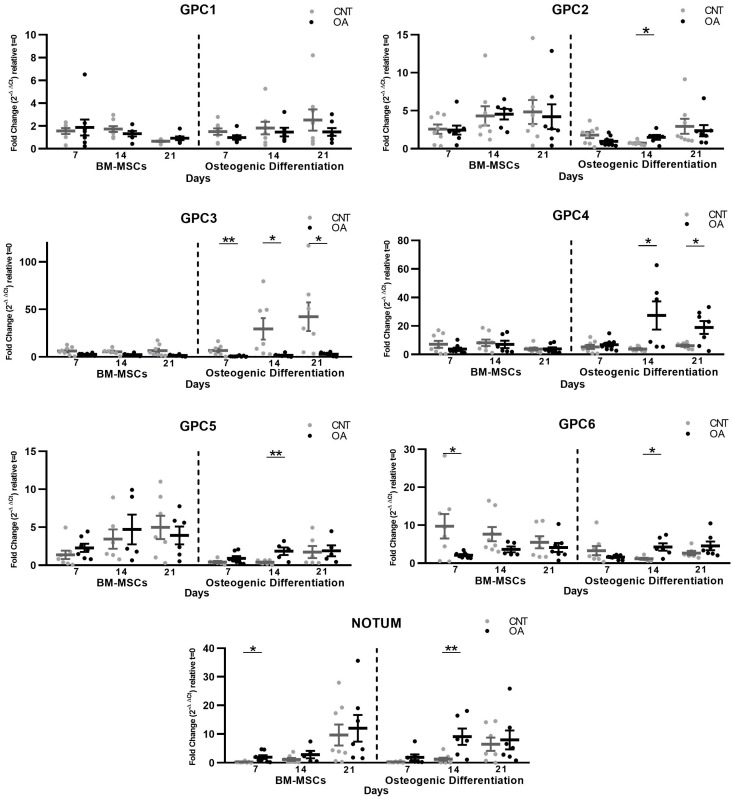
Gene expression of Glypicans and Notum measured by qPCR at 7, 14 and 21 days of culture in patients with Osteoarthritis (*n* = 8) and controls (*n* = 8). Relative gene expression was normalized to the housekeeping genes β-actin and RNA18S. The data were calculated with the fold change method (2^−ΔΔCt^) relative to *t* = 0. T-tests were used to compare the groups: * *p* < 0.05, ** *p* < 0.01. All bar graphs are presented as mean ± SEM. In OA BM-MSCs, a decrease in the expression of GPC6 and an increase in Notum were found. During osteogenic differentiation, upregulation of the mRNA levels of GPC2, GPC4, GPC5, GPC6 and Notum and downregulation of GPC3 were found in OA patients.

Our results showed a decrease in the expression of GPC6 in the OA group compared to the control group (*p* = 0.0471) and an increase in Notum (*p* = 0.0156) at 7 days of BM-MSC culture. When inducing osteogenic differentiation, upregulation of the mRNA levels of GPC2 (*t* = 14 d; *p* = 0.034), GPC4 (*t* = 14 d, *p* = 0.025; *t* = 21 d, *p* = 0.017), GPC5 (*t* = 14 d, *p* = 0.007), GPC6 (*t* = 14 d, *p* = 0.013) and Notum (*t* = 14 d, *p* = 0.009) was observed in OA patients. During the 21 days of differentiation, GPC3 was downregulated in patients with OA compared to the control group (*t* = 7 d, *p* = 0.009; *t* = 14 d, *p* = 0.045; *t* = 21 d, *p* = 0.036) (Figure 2).

#### 3.2.2. Protein Levels in Supernatant

In the BM-MSCs of the OA group compared to the control group, GPC3 protein levels were higher during the 21 days of culture (*t* = 0, 7, 14, 21, *p* < 0.0001). In addition, lower protein levels of GPC5 (*t* = 7 d, *p* = 0.0006) and Notum (*t* = 0, *p* = 0.0497) were found.

**Figure 3:**
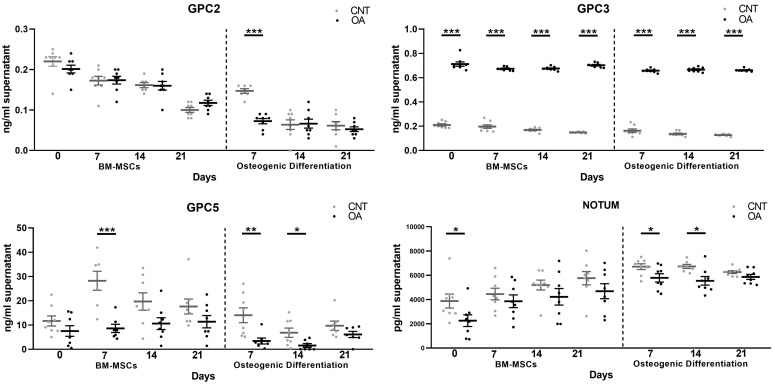
Protein levels of Glypicans and Notum determined by ELISA kit at 0, 7, 14 and 21 days of BM-MSC and osteoblast culture in patients with Osteoarthritis (*n* = 8) and controls (*n* = 8). T-tests were used to compare the groups: * *p* < 0.05, ** *p* < 0.01, *** *p* < 0.001. All bar graphs are presented as mean ± SEM. In OA BM-MSCs, a decrease in GPC5 and Notum and an increase in GPC3 were found. During osteogenic differentiation, a decrease in GPC2, GPC5 and Notum and an increase in GPC3 were observed in OA patients. GPC1, GPC4 and GPC6 were not found in soluble form.

During all osteogenic differentiation experiments, an increase in GPC3 was found (*p* < 0.0001). Furthermore, low levels of GPC2 (*t* = 7 d, *p* < 0.0001), GPC5 (*t* = 7 d, *p* = 0.0089; *t* = 14 d, *p* = 0.0311) and Notum (*t* = 7, *p* = 0.0432; *t* = 14 d, *p* = 0.0136) were observed. Soluble forms of GPC1, GPC4 and GPC6 were not found in any of the cell cultures (Figure 3).

#### 3.2.3. Summary of BM-MSC and Osteoblast Results

Table 1 shows the results obtained regarding gene expression and protein levels during the cell culture of BM-MSCs and differentiation into osteoblasts.

## 4. Discussion

Globally, Osteoarthritis is a leading cause of disability, with a dynamically increasing prevalence due to an aging population and increased risk factors. This joint disease is characterized by a loss of articular cartilage and a progressive thickening of the subchondral bone. Current treatments are mainly drugs to relieve symptoms and joint replacement surgery in cases where patients’ daily lives are severely impacted [4]. Therefore, one of the main objectives of healthcare systems today is to find an effective treatment.

Therapeutic interventions for Osteoarthritis have particularly focused on cartilage degradation. However, in recent years, increasing evidence has shown that alterations in the subchondral bone have an integral role in the process of OA [7]. Among the molecular alterations involved in OA and related to bone formation, excessive activation of the Wnt/β-catenin signaling pathway has been found [18]. The activation of this pathway promotes osteogenic differentiation preferentially, inhibiting chondrogenic differentiation [9]. This provides an explanation for why increased bone mass and cartilage loss occur in OA. Mediators related to the canonical Wnt/β-catenin signaling pathway promote the activation and deactivation of the pathway [15]. Despite their importance, the alterations of some mediators related to the Wnt signaling pathway in the context of Osteoarthritis are still unknown. By improving understanding of the deregulations in bone metabolism caused by OA, treatments can be proposed to restore homeostasis, modulating the active osteogenic metabolism and, as a possible consequence, the regulation of chondrogenic metabolism. Therefore, the objective of this study was to analyze the levels of Glypicans and Notum, modulators of the Wnt pathway, in patients with OA compared to healthy donors. These molecules were studied in patient plasma and BM-MSCs and during osteogenic differentiation.

Our results show lower circulating levels of Glypican 5 and Notum in patients with OA. These results agree with the levels of these proteins that were identified in cells. In BM-MSCs, both Glypican 5 and Notum were found in lower amounts in OA patients compared to cells from healthy patients. During osteogenic differentiation, it was found that the levels were also decreased. This demonstrates that the deregulation of these two components at the cellular level can be confirmed in patient plasma. For this reason, Glypican 5 and Notum could be promising candidates for biomarkers of osteoarthritis.

On the other hand, Glypicans in the extracellular matrix are negative regulators of the Wnt β-Catenin signaling pathway [15]. Notum also acts as a negative regulator of the Wnt pathway [16]. Our findings on the soluble forms of Glypican 5 and Notum are very important in this sense. The alterations found are intrinsic to the BM-MSCs of patients with osteoarthritis, since these two molecules were decreased. It is possible that this alteration contributes to the activation of the Wnt/β-Catenin signaling pathway found in OA.

Interestingly, Glypican 3, homologous to Glypican 5, showed an increase in the protein levels throughout the culture of the mesenchymal cells. In a study with GPC3 knockout mice, it led to the inhibition of the non-canonical Wnt/JNK signaling pathway. However, it caused the activation of canonical Wnt/β-Catenin signaling [19]. Although it has been proposed that membrane-bound GPC3 is closely related to the activation of the canonical Wnt/β-Catenin signaling pathway [20], it is likely that the complete lack of Glypican 3 activates other processes so that the canonical pathway remains active. The excessive increase in soluble Glypican 3 found in our study, together with the decreased production of mRNA, indicates that Glypican 3 may not be practically membrane-bound and that it contributes to the activation of the canonical Wnt pathway.

There is some evidence that Glypican 5 inhibits the Wnt/β-catenin pathway, such as a study carried out on prostate cancer in which GPC5 was poorly expressed in the cancer cell lines. Upregulation inhibited cell proliferation in vitro and invasion and attenuated tumor growth in vivo. Furthermore, the authors found that GPC5 overexpression inhibited epithelial–mesenchymal transition (EMT) and Wnt/β-catenin signaling [21]. In our study, during osteogenesis we identified the overexpression of GPC5, perhaps as a negative regulatory element of the Wnt pathway.

In the context of Osteoarthritis and Glypican 3, a recent study evaluated GPC3 levels in the synovial fluid of patients with knee OA compared to controls. An accumulation of GPC3 was found in the synovial fluid [22]. These findings, together with the excessive amount of GPC3 in the extracellular medium that we found in BMCs and osteoblasts, could indicate that a localized accumulation of GPC3 occurs in OA throughout the joint. In this same study, a decrease in GPC3 seen in the plasma of patients with the disease was associated and inversely associated with radiographic severity [23]. Our data were not significant regarding GPC3 in blood circulation. It would be necessary to study a larger number of participants, including an analysis of Glypicans 1 to 6 and Notum, to relate it to the severity of the disease.

During osteogenic differentiation, our results on the soluble form of Glypicans showed the same existing alterations as in mesenchymal stem cells regarding GPC3, GPC5 and Notum. On days 7 and 14 of differentiation, the levels of GPCs 5 and 2 in the extracellular medium were decreased. In our study of the gene expression of these components, the results showed the overexpression of GPCs 2 and 5. For its part, GPC3 was excessively accumulated throughout the culture in the extracellular medium, as found in MSCs, and the mRNA data showed an upregulation of GPC3. This proves that the gene expression and protein level results are inversely related in the extracellular medium during differentiation towards osteoblasts. It could be that during osteogenic differentiation, an attempt is made to regulate existing alterations.

In MSCs and during osteogenesis, Notum showed an increased production and a decreased presence in the extracellular matrix. Wnt activation stimulates Notum production as a regulatory mechanism of the Wnt/β-Catenin signaling pathway [23]. Furthermore, this enzyme is related to bone formation since Notum expression is elevated in cultured osteoblasts and not in osteoclasts [24]. Our results are consistent with the evidence, as they show a high production of Notum at the cellular level in patients with OA, an indirect indication that the Wnt pathway is active and related to the bone growth characteristic of this disease.

GPCs 1, 4 and 6 were not present in soluble form in the cultures under either condition, nor in patients nor healthy donors. However, we found that the gene expressions of GPCs 4 and 6 were both deregulated. The specific function of each is not fully known, so their involvement remains to be elucidated. However, due to the great interest in the involvement of Glypicans in different diseases, there is further evidence of their role. In the case of Glypican 6, a previous study determined that it binds to Wnt3a, even more specifically than Glypican 3, and inhibits canonical Wnt signaling [20] despite what might be expected, since membrane-bound Glypicans are supposed to facilitate the activation of the pathway. In our study, we found that OA mesenchymal cells produced lower amounts of mRNA in GPC6, so it would not contribute to deactivating the pathway. In a possible attempt at regulation, the data showed an upregulation in GPC6 gene expression during osteogenic differentiation.

In our findings, there appears to be a functional relationship between GPC3 and NOTUM. In both MSCs and osteoblasts, we found an upregulation in gene expression of Notum accompanied by a decrease in the extracellular medium, while the opposite occurred in Glypican 3. In a study on colorectal cancer, the authors observed a relationship between Notum and GPC3 in which the production of mRNA and protein was inversely correlated [25]. In fact, this relationship was observed in another previous investigation that proposed that Notum could act as a phospholipase enzyme and cleave the GPI anchor, which keeps Glypicans attached to the cell membrane [16]. However, a recent investigation demonstrated the extracellular role that Notum played and what its interactions were. The authors verified that Notum is not a Glypican-specific phospholipase as had been deduced, but that it is a Wnt-specific deacylating protein with a preference for long-chain, cis-unsaturated lipids. Therefore, it eliminates an essential palmitoleate fraction from Wnts and deactivates the signaling pathway. Notum does not act on Glypicans enzymatically but binds to them, and its sulfated GAG chains of Glypicans probably mediate the interaction of Notum with Wnts [17]. Therefore, although there is evidence of the relationship between Notum and Glypican 3 in different studies, it is not currently known what the specific mechanism by which they may be related is.

It could be interesting to relate our results to the severity of the disease, age or gender. On the other hand, in vitro tests do not fully reflect the complexity of joints, so some in vivo studies would also be appropriate.

In conclusion, the Wnt/beta-catenin signaling pathway is crucial in the development and homeostasis of various tissues and organs in multicellular organisms, and its dysregulation is implicated in several diseases, including osteoarthritis [10,11,12,13]. Glypicans are glycosylphosphatidylinositol (GPI)-anchored membrane proteoglycans that have been shown to interact with components of the Wnt pathway, modulating its activity [14,15]. On the other hand, Notum is an enzyme that negatively regulates Wnt signaling by deacetylating the Wnt ligand, thereby inhibiting its activity [16,17]. Theoretically, the activation of Notum or the deactivation of glypicans could have a negative effect on the Wnt/beta-catenin pathway, which could be beneficial in conditions where this pathway is hyperactive, such as osteoarthritis. However, to confirm the functional impact of these approaches in in vivo models and evaluate their therapeutic potential, additional experimental studies would be needed. These could include trials in animal models, such as transgenic mice or xenografts, to investigate the effects of Notum activation or glypican deactivation on disease progression and treatment response. Ultimately, a detailed understanding of the interaction between these regulators and the Wnt/beta-catenin pathway, as well as their impact on disease, would be crucial for the development of potential targeted therapies.

## Figures and Tables

**Table 1:** Summary of Glypicans 1 to 6 (GPCs) and Notum results in BM-MSCs and osteoblasts in Osteoarthritis (OA) patients. Gene expression measured by qPCR and protein levels determined by ELISA.

		GPC1	GPC2	GPC3	GPC4	GPC5	GPC6	Notum
BM-MSCs	Gene expression (qPCR)	---	---	---	---	---	↓↓↓	↑↑↑
	Protein levels (ELISA)	---	---	↑↑↑	---	↓↓↓	---	↓↓↓
Osteogenic differentiation	Gene expression (qPCR)	---	↑↑↑	↓↓↓	↑↑↑	↑↑↑	↑↑↑	↑↑↑
	Protein levels (ELISA)	---	↓↓↓	↑↑↑	---	↓↓↓	---	↓↓↓

↑↑↑—very high; ↓↓↓—very low.

## Data Availability

The data presented in this study are available on request from the corresponding author.

## References

[1] Palazzo C., Nguyen C., Lefevre-Colau M.-M., Rannou F., Poiraudeau S. (2016). Risk factors and burden of osteoarthritis. Ann. Phys. Rehabil. Med..

[2] Martel-Pelletier J., Barr A.J., Cicuttini F.M., Conaghan P.G., Cooper C., Goldring M.B., Goldring S.R., Jones G., Teichtahl A.J., Pelletier J.-P. (2016). Osteoarthritis. Nat. Rev. Dis. Prim..

[3] Hunter D.J., McDougall J.J., Keefe F.J. (2008). The Symptoms of Osteoarthritis and the Genesis of Pain. Rheum. Dis. Clin. N. Am..

[4] Hunter D.J., Felson D.T. (2006). Osteoarthritis. BMJ.

[5] Uccelli A., Moretta L., Pistoia V. (2008). Mesenchymal stem cells in health and disease. Nat. Rev. Immunol..

[6] Murphy J.M., Dixon K., Beck S., Fabian D., Feldman A., Barry F. (2002). Reduced chondrogenic and adipogenic activity of mesenchymal stem cells from patients with advanced osteoarthritis. Arthritis Rheum..

[7] Tat S.K., Lajeunesse D., Pelletier J.-P., Martel-Pelletier J. (2010). Targeting subchondral bone for treating osteoarthritis: What is the evidence?. Best Pract. Res. Clin. Rheumatol..

[8] Duan P., Bonewald L. (2016). The role of the wnt/β-catenin signaling pathway in formation and maintenance of bone and teeth. Int. J. Biochem. Cell Biol..

[9] Escobar-Gómez F., Jódar E., Hawkins F. (2009). Receptor Wnt: Fisiología, fisiopatología y potenciales nuevas dianas terapéuticas. Rev. Española Enfermedades Metabólicas Óseas.

[10] Corr M. (2008). Wnt–β-catenin signaling in the pathogenesis of osteoarthritis. Nat. Clin. Pract. Rheumatol..

[11] Tornero-Esteban P., Peralta-Sastre A., Herranz E., Rodríguez-Rodríguez L., Mucientes A., Abásolo L., Marco F., Fernández-Gutiérrez B., Lamas J.R. (2015). Altered Expression of Wnt Signaling Pathway Components in Osteogenesis of Mesenchymal Stem Cells in Osteoarthritis Patients. PLoS ONE.

[12] Blom A.B., Brockbank S.M., van Lent P.L., van Beuningen H.M., Geurts J., Takahashi N., van der Kraan P.M., van de Loo F.A., Schreurs B.W., Clements K. (2009). Involvement of the Wnt signaling pathway in experimental and human osteoarthritis: Prominent role of Wnt-induced signaling protein 1. Arthritis Rheum..

[13] Held A., Glas A., Dietrich L., Bollmann M., Brandstädter K., Grossmann T., Lohmann C., Pap T., Bertrand J. (2018). Targeting β-catenin dependent Wnt signaling via peptidomimetic inhibitors in murine chondrocytes and OA cartilage. Osteoarthr. Cartil..

[14] Filmus J., Capurro M., Rast J. (2008). Glypicans. Genome Biol..

[15] Boudin E., Fijalkowski I., Piters E., Van Hul W. (2013). The role of extracellular modulators of canonical Wnt signaling in bone metabolism and diseases. Semin. Arthritis Rheum..

[16] Traister A., Shi W., Filmus J. (2008). Mammalian Notum induces the release of glypicans and other GPI-anchored proteins from the cell surface. Biochem. J..

[17] Kakugawa S., Langton P.F., Zebisch M., Howell S.A., Chang T.-H., Liu Y., Feizi T., Bineva G., O’reilly N., Snijders A.P. (2015). Notum deacylates Wnt proteins to suppress signalling activity. Nature.

[18] Wang Y., Fan X., Xing L., Tian F. (2019). Wnt signaling: A promising target for osteoarthritis therapy. Cell Commun. Signal..

[19] Song H.H., Shi W., Xiang Y.-Y., Filmus J. (2005). The Loss of Glypican-3 Induces Alterations in Wnt Signaling. J. Biol. Chem..

[20] Capurro M., Martin T., Shi W., Filmus J. (2014). Glypican-3 binds to frizzled and plays a direct role in the stimulation of canonical Wnt signaling. J. Cell Sci..

[21] Sun Y., Xu K., He M., Fan G., Lu H. (2018). Overexpression of Glypican 5 (GPC5) Inhibits Prostate Cancer Cell Proliferation and Invasion via Suppressing Sp1-Mediated EMT and Activation of Wnt/β-Catenin Signaling. Oncol. Res. Featur. Preclin. Clin. Cancer Ther..

[22] Udomsinprasert W., McConachie E., Ngarmukos S., Theerawattanapong N., Tanavalee A., Honsawek S. (2021). Plasma and Joint Fluid Glypican-3 Are Inversely Correlated with the Severity of Knee Osteoarthritis. Cartilage.

[23] Torisu Y., Watanabe A., Nonaka A., Midorikawa Y., Makuuchi M., Shimamura T., Sugimura H., Niida A., Akiyama T., Iwanari H. (2008). Human homolog of NOTUM, overexpressed in hepatocellular carcinoma, is regulated transcriptionally by β-catenin/TCF. Cancer Sci..

[24] Movérare-Skrtic S., Nilsson K.H., Henning P., Funck-Brentano T., Nethander M., Rivadeneira F., Nunes G.C., Koskela A., Tuukkanen J., Tuckermann J. (2019). Osteoblast-derived NOTUM reduces cortical bone mass in mice and the NOTUM locus is associated with bone mineral density in humans. FASEB J..

[25] De Robertis M., Arigoni M., Loiacono L., Riccardo F., Calogero R.A., Feodorova Y., Tashkova D., Belovejdov V., Sarafian V., Cavallo F. (2015). Novel insights into Notum and glypicans regulation in colorectal cancer. Oncotarget.

